# Authors’ response to Letter to the Editor “Cardiac lymphoid hyperplasia is same as cardiac nodularity‐like appearance”

**DOI:** 10.1002/deo2.106

**Published:** 2022-03-25

**Authors:** Kyoichi Adachi, Norihisa Ishimura, Shunji Ishihara

**Affiliations:** ^1^ Health Center Shimane Environment and Health Public Corporation Shimane Japan; ^2^ Second Department of Internal Medicine Shimane University Faculty of Medicine Shimane Japan

We thank Drs. Nishizawa, Yoshida, and Toyoshima for their interest in our manuscript,^1^ and for providing important comments. In that study, we analyzed results obtained from 1884 patients with Barrett's epithelium and found that endoscopic findings of lymphoid hyperplasia were well correlated with the status of *Helicobacter pylori (H. pylori)* infection. In addition, the duration after *H. pylori* eradication was demonstrated to be negatively correlated with its prevalence. Nishizawa et al.^2^ noted that “endoscopic findings of cardiac lymphoid hyperplasia” were the same as findings of a “cardiac nodularity‐like appearance” noted in their previous studies.^3^, ^4^ Furthermore, they demonstrated that “cardiac nodularity‐like appearance” was associated with *H. pylori* infection and was improved by *H. pylori* eradication.^3^, ^4^ We agree that endoscopic findings demonstrating “cardiac lymphoid hyperplasia” are the same as a “cardiac nodularity‐like appearance” and recommend that the terms be unified. In our manuscript, we used the term “cardiac lymphoid hyperplasia” to show attention to the histological finding itself in that study,^1^ rather than endoscopic appearance. The significance of lymphoid hyperplasia in a cardiac portion should be clarified in a future study, as antral nodular gastritis is a well‐known risk factor related to gastric cancer incidence.

## CONFLICT OF INTEREST

Norihisa Ishimura is an Associate Editor of DEN Open. The rest of the authors do not have any conflict of interest.

## FUNDING INFORMATION

None.
